# A Cell-Penetrating Peptide Improves Anti-HER2 Single-Chain Variable Fragment Internalization and Antitumor Activity against HER2-Positive Breast Cancer In Vitro and In Vivo

**DOI:** 10.3390/molecules29061247

**Published:** 2024-03-11

**Authors:** Junmin Li, Yanting Zhou, Zhuowei Su, Xue Li, Lei Zhang, Shan Li

**Affiliations:** 1 MOE International Joint Laboratory for Synthetic Biology and Medicines, School of Biology and Biological Engineering, South China University of Technology, Guangzhou 510006, China; lijunmin5760@163.com (J.L.); laurachow0828@163.com (Y.Z.); snow0377@163.com (X.L.); lzhangce@scut.edu.cn (L.Z.); 2 School of Biology and Food Science, Shangqiu Normal University, Shangqiu 476000, China; 3 Guangdong Provincial Hospital of Chinese Medicine, The Second Affiliated Hospital of Guangzhou, University of Chinese Medicine, Guangzhou 510006, China; szw2003@126.com; 4 Nanyang Medical College, Nanyang 473001, China; 5 NMPA Key Laboratory for Quality Control of Blood Products, Guangdong Institute for Drug Control, Guangzhou 510663, China

**Keywords:** HER2-positive breast cancer, scFv, leader peptide-scFv, internalization, antitumor activity

## Abstract

Cell-penetrating peptides (CPPs) are invaluable tools for delivering various substances into cells by crossing biological membranes. However, the effects of cell-penetrating peptide fusion proteins on the biological activity of antibodies remain to be fully understood. Here, we engineered a recombinant protein, LP-scFv, which combines the single-chain variable region of anti-human epidermal growth factor receptor-2 with a novel and non-oxic cell-penetrating peptide as a leader peptide. The introduction of this leader peptide led to a more than twofold increase in the internalization efficiency of the single-chain antibody, as confirmed using microscopic analysis and flow cytometry. The effects of the single-chain antibodies and LP-scFv on cell viability were evaluated using the MTT assay. Both the single-chain antibodies and LP-scFv reduced the viability of BT474 and NCI-N87 cells in a dose-dependent manner while exhibiting minimal toxicity towards MCF-7 and MCF-10A cells. Further investigation into LP-scFv’s mechanism revealed that the induced leader peptide does not alter the MAPK-ERK1/2 and PI3K/AKT pathways of single-chain antibodies. An enhanced antitumor activity was also confirmed in an NCI-N87 tumor xenograft model in mice with a reduction of 45.2% in tumor growth inhibition (vs. 23.1% for scFv) with a 50 mg/kg dose after orthotopic injection administration, which was equivalent to that of trastuzumab (vs. 55.7% for trastuzumab). Overall, these results indicate that LP-scFv exhibits significant permeation activity in HER2-positive cells to enhance the intracellular dose effect on antitumor activity in vitro and in vivo. This research lays the foundation for designing novel antibody-based therapies for cancer.

## 1. Introduction

Breast cancer, a clinically and molecularly heterogeneous malignancy, is a leading cause of cancer-associated mortality in women. In 2020, it accounted for 2.26 million new cases globally, representing 11.7% of all new cancer cases [1]. Clinical studies have identified the human epidermal growth factor receptor 2 (HER2) as a crucial target in HER2-positive metastatic breast cancer therapy [2]. HER2-targeted therapies, particularly antibody-related drugs, have significantly enhanced the prognosis of HER2-positive breast cancer [3]. However, HER2-positive breast cancer patients often exhibit high primary or acquired drug resistance at both primary and metastatic sites [4,5]. This resistance partly stems from the characteristics of monoclonal antibody drugs, a large molecular weight (about 150 kDa), a low internalization efficiency, slow tumor penetration, a low delivery dose to the tumor site (<1%), and prolonged in vivo clearance (several weeks), potentially causing untargeted cytotoxicity [6,7]. Promoting the internalization and tissue penetration of antibodies is a key research focus to improve the efficacy of antibody-based drugs.

The singl-chain variable fragment (scFv), lacking the Fc segment found in IgG antibodies, has a smaller molecular weight, which facilitates tissue penetration and access to the hidden epitopes. Moreover, an scFv reduces potential immunogenicity and can be cost-effectively produced in large quantities using microbial expression systems [8]. However, the cell membrane, a semi-permeable barrier, primarily allows for the rapid passage of small, uncharged molecules such as water and steroid hormones. Hydrophilic macromolecules larger than 500–1000 Da exhibit poor membrane permeability [9]. Therefore, the cell membrane presents a significant challenge to developing medium- to large-scale drugs based on peptides, proteins, antibodies, and nucleic acids. With a molecular weight of about 30 kDa, an scFv is considered a macromolecule relative to the cell membrane, resulting in a low internalization efficiency. Enhancing scFv internalization is crucial for improving its therapeutic efficacy [10].

Cell-penetrating peptides (CPPs), oligopeptide carriers capable of effectively traversing cell membranes to deliver diverse substances (including peptides, DNA, proteins, liposomes, and drugs), have been widely studied [11,12,13]. For example, cationic TAT peptides rich in arginine and lysine efficiently transport antibodies, antibody fragments, and nanoparticles into cells [14,15]. The amphipathic model amphipathic peptide (MAP) with an alpha (α)-helix structure can spontaneously insert into the lipid monolayer and then induce cellular transport of peptides or small-molecule proteins [16,17]. Hydrophobic peptide gH625 is a membrane-perturbing domain, which interacts with biological membranes and is able to transport different cargoes into the cytoplasm [18]. Due to CPPs’ non-specific mediation, coupling monoclonal antibodies to CPP-functionalized carriers has emerged as an effective strategy for enhancing targeted delivery, with significant recent advancements [19,20,21]. Lacasse et al. were the first to report a cholic-acid-linked conjugate, combining a nuclear localization signal sequence with trastuzumab-emtansine (T-DM1). This conjugate improved the nuclear localization efficiency and significantly improved the cytotoxic effect of T-DM1 against HER2-positive breast cancer [22].

Coupling a CPP with an scFv increases the specificity of the CPP and improves scFv’s internalization efficiency. Mitochondrial precursor proteins, synthesized by cytoplasmic ribosomes, possess a 20–30-amino-acid signal or leader peptide at the N-terminus. This peptide forms a positively charged amphiphilic α-helix, essential for efficient protein transport [23,24,25,26]. This study uses a leader peptide, LP, with an amphiphilic helix structure derived from cytochrome oxidase, targeting the mitochondrial inner membrane [27]. The sequence of the LP is YHMMLSALARPASAALRRSFSTSAQNN, and its molecular weight is about 3 kDa. The anti-HER2 scFv was identified in our laboratory using a phage display screening technique, and a patent for the fusion protein LP-scFv has been granted (CN Patent No. 201710683659.5) (http://epub.cnipa.gov.cn/Index, accessed on 25 March 2022).

This study engineered a recombinant LP-scFv, combining an LP and anti-HER2 scFv, to investigate antibody internalization and tissue penetration. Subsequently, the in vivo and in vitro mechanisms of LP-scFv were explored. LP-scFv was found to promote scFv endocytosis and enhance its antitumor activity by increasing the intracellular dose effect. Further analysis indicated that LP-scFv induced cytotoxic activity and apoptosis in the scFv through the MAPK-ERK1/2 and PI3K/AKT pathways. These findings offer valuable insights for designing small-molecular-antibody-based therapies. Additionally, these results suggest that improving the internalization efficacy in macromolecular antibodies requires novel approaches.

## 2. Results and Discussion

### 2.1. Construction, Expression, and Purification of Recombinant scFv and LP-scFv

The purification of scFv, LP-scFv, scFv-EGFP, and LP-scFv-EGFP proteins was confirmed via SDS-PAGE and Western blotting. The results demonstrated that these recombinant proteins exhibited distinct bands, indicating a high purity (>90%), with molecular weights of approximately 31 kDa, 35 kDa, 55 kDa, and 58 kDa, respectively (Supplementary Materials Figure S1).

The binding affinities of the scFv and LP-scFv to HER2 were measured using label-free biolayer interferometry (BLI) [28,29], as shown in Figure S2. Table 1 presents the calculated association (*k*_on_) and dissociation (*k*_off_) rate constants, along with the equilibrium dissociation constants (*K*_D_ = *k*_off_/*k*_on_). These values indicate comparable kinetic behaviors for both the scFv and LP-scFv in their interaction with HER2, implying that LP fusion did not compromise antigen-binding capabilities.

### 2.2. ScFv and LP-scFv Binding and Internalization

LP-EGFP was initially expressed in *E. coli* and purified using nickel affinity chromatography. This process yielded a distinct ~31 kDa band in both SDS-PAGE and Western blotting (Figure S1). Following a 24 h incubation of BT474 (a human breast ductal carcinoma cell line), NCI-N87 (a human gastric cancer cell line), MCF-7 (a human breast cancer cell line), MCF-10A (a human normal breast epithelial cell line), and human hepatic stellate LX2 cells with 500 nM LP-EGFP, flow cytometry was used to measure the fluorescence intensity in each cell line. This analysis aimed to assess the impact of LP coupling on the internalization of the macromolecular EGFP. Figure S3 demonstrated that, compared to EGFP alone, LP-EGFP exhibited a significantly improved internalization efficiency. This finding suggests that LP enhanced the internalization of the EGFP protein across different cell types without selectivity.

**Figure 1:**
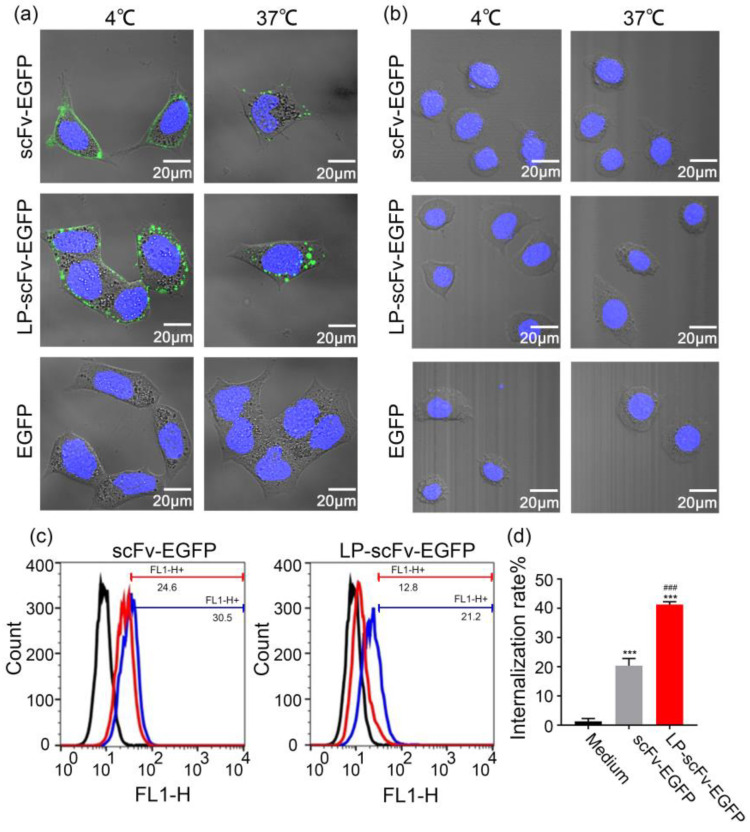
The internalization of scFv-EGFP and LP-scFv-EGFP in HER2-positive BT474 cells and HER2-negative MCF-7 cells. Confocal images of internalization after scFv-EGFP and LP-scFv-EGFP binding to BT474 cells (**a**) and MCF-7 cells (**b**) with Hoechst 33342 used for nuclear contrast (600×; scale bar, 20 μm). (**c**) The binding and endocytosis ability of scFv-EGFP or LP-scFv-EGFP to BT474 cells via flow cytometry. Black line: control; blue line: 4 °C; red line: 37 °C. (**d**) The internalization efficacy of scFv-EGFP or LP-scFv-EGFP in BT474 cells. *** *p* < 0.001, vs. medium; ^###^ *p* < 0.001, vs. scFv-EGFP.

Internalization of cell-surface-bound scFv and LP-scFv fragments was assessed using established methods [30,31]. As shown in Figure 1a, scFv-EGFP and LP-scFv-EGFP were specifically bound to HER2-positive BT474 cells at 4 °C, indicated by pronounced membrane fluorescence. Cells were then washed at 4 °C to remove unbound proteins, after which cells were incubated for 2 h at 37 °C; increased cytoplasmic fluorescence demonstrated their internalization. Confocal microscopy (Figure 1b) confirmed the binding and internalization of both scFv-EGFP and LP-scFv-EGFP in HER2-positive BT474 cells, with no significant fluorescence observed in HER2-negative MCF-7 cells. EGFP alone showed neither binding nor internalization in BT474 and MCF-7 cells. Flow cytometry was used to quantify the internalization efficacy of scFv-EGFP and LP-scFv-EGFP by measuring the fluorescence shifts on the cell surface relative to control cells (Figure 1c). The results showed that LP-scFv-EGFP internalization (41.2% ± 1.0%) was about twice that of scFv-EGFP (20.4% ± 2.4%), indicating that the LP significantly enhanced scFv-EGFP internalization in BT474 cells (Figure 1d).

**Figure 2:**
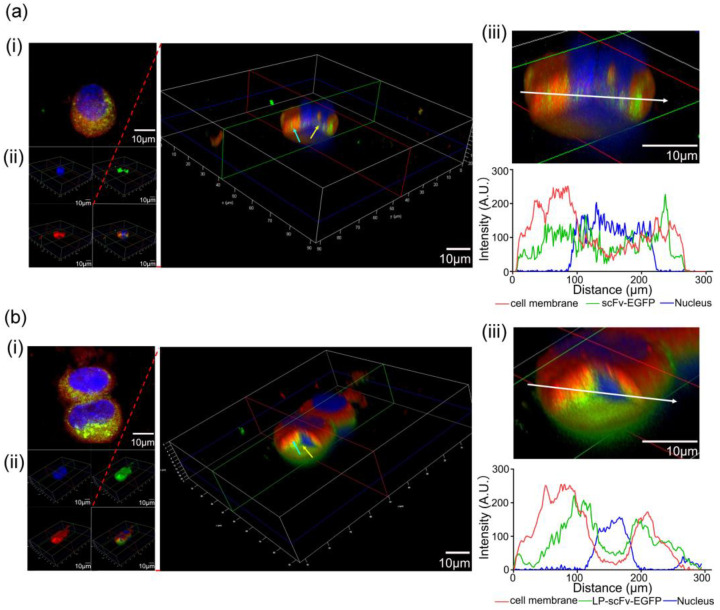
The confocal 3D section of BT474 cells treated with scFv-EGFP (**a**) and LP-scFv-EGFP (**b**). Example Z-projection image and 3D section of the side view of a BT474 cell stained for nucleus, cell membrane, scFv-EGFP (**a**(**i**,**ii**)) and LP-scFv-EGFP (**b**(**i**,**ii**)). Quantitative fluorescence analysis of the cell membrane, nucleus, scFv-EGFP (**a**(**iii**)), and LP-scFv-EGFP (**b**(**iii**)) along the front-to-back axis (white arrow) of BT474 cells.

To confirm the endocytosis of scFv-EGFP and LP-scFv-EGFP into BT474 cells, three-dimensional (3D) models were created. BT474 cells, treated with scFv-EGFP or LP-scFv-EGFP and stained with Hoechst 33342 and far-infrared DiD, were analyzed using confocal microscopy. Both scFv-EGFP (Figure 2a(i)) and LP-scFv-EGFP (Figure 2b(i)) were observed near the nucleus (blue), with LP-scFv-EGFP showing a more pronounced distribution. Three-dimensional sections of the BT474 cells showed that a part of scFv-EGFP or LP-scFv-EGFP (green; cyan arrow) was embedded in the cell membrane (red), indicating that this part of the protein bound to the HER2 antigen on the cell membrane and had not been endocytosed into the cell. The other part of the scFv-EGFP or LP-scFv-EGFP protein (green; yellow arrow) was apart from the cell membrane (red) and had access to the nucleus (blue), which indicated that this part of the scFv-EGFP or LP-scFv-EGFP protein had entered the cell (Figure 2a(ii),b(ii)). More importantly, most LP-scFv-EGFP (green; yellow arrow) was between the cell membrane (red) and the nucleus (blue), which indicated that most of the LP-scFv-EGFP protein was endocytosed into the cell. Quantitative fluorescence analysis of BT474 cells (Figure 2a(iii),b(iii)) showed 44.2% overlap of scFv-EGFP with the cell membrane (red), and 21.5% for LP-scFv-EGFP, indicating internalization of these proteins, particularly LP-scFv-EGFP. Laser confocal microscopy results suggest that coupling an LP with the scFv maintained specificity and improved scFv internalization.

### 2.3. Cytotoxicity and Apoptosis Induction

**Figure 3:**
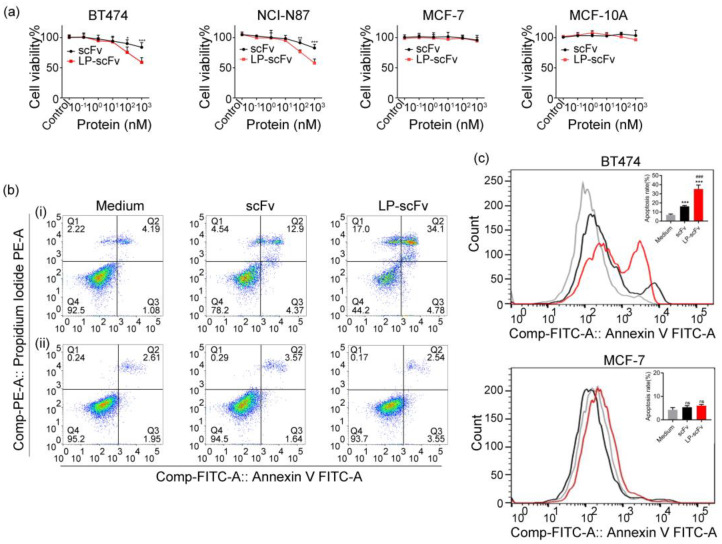
ScFv and LP-scFv inhibited the proliferation and induced the apoptosis of BT474 and MCF-7 cells. (**a**) The viability of BT474, MCF-7, NCI-N87, and MCF-10A cells was detected via an MTT assay. * *p* < 0.05, ** *p* < 0.01, *** *p* < 0.001, vs. scFv group. (**b**) The apoptosis of BT474 (**i**) and MCF-7 (**ii**) cells treated with scFv or LP-scFv was measured via flow cytometry. (**c**) Quantification of the apoptosis rate (including early and late apoptosis) for cells treated as in (**b**). *** *p* < 0.001 vs. medium, ^###^ *p* < 0.001 vs. scFv group. ns, no significance.

Experiments combining confocal microscopy and flow cytometry demonstrated that LP-scFv had no effect on the specific binding of the scFv to the HER2 antigen and increased the internalization efficiency of the scFv by about twofold. Subsequently, the impact of scFv and LP-scFv on cell viability was assessed using MTT assays and flow cytometry. Initially, the LP peptide, synthesized commercially, was co-cultured with BT474, NCI-N87, MCF-7, and MCF-10A cells at varying concentrations (0, 1, 5, 10, 25, 50 μg/mL) for 72 h, showing no significant effect on cell viability (Figure S4). Furthermore, the effect of the scFv and LP-scFv on the viability of MCF-10A, BT474, NCI-N87, and MCF-7 cells was evaluated, and the result indicated that both scFv and LP-scFv proteins were able to impair BT474 and NCI-N87 cell viability in a dose-dependent fashion, with LP-scFv exhibiting a superior anti-proliferative activity relative to the scFv (Figure 3a). In contrast, neither fragment could induce significant toxicity in MCF-7 and MCF-10A cells. To confirm apoptosis induction by scFv and LP-scFv treatments, flow cytometry was used to measure early (Q2; Annexin V^+^/PI^−^) and late (Q3; Annexin V^+^/PI^+^) apoptotic cells in BT474 and MCF-7 cells. Results showed that before treatment, most BT474 cells were alive (92.5% ± 0.5%). A total of 38.9% ± 1.6% of cells were apoptotic following LP-scFv treatment, compared to 17.3% ± 2.3% after scFv treatment, indicating LP-scFv’s higher efficacy in inducing apoptosis. In contrast, both the scFv and LP-scFv induced minimal apoptosis in MCF-7 cells, with rates of 5.2% ± 0.9% and 6.1% ± 0.7%, respectively (Figure 3b,c).

**Figure 4:**
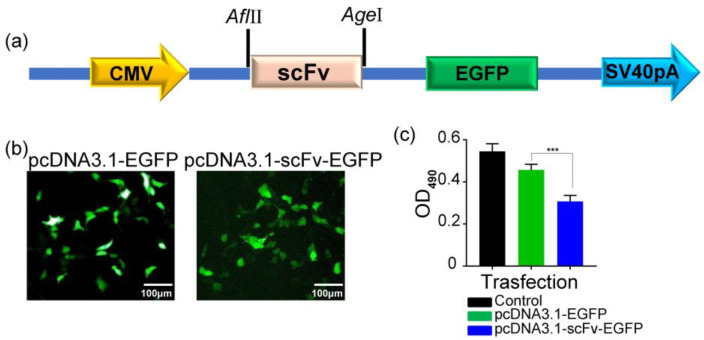
The effect of pcDNA3.1-scFv-EGFP transfection on the activity of HER2-positive cells BT474. (**a**) The pcDNA3.1-scFv-EGFP plasmid was used in this study. (**b**) Fluorescence microscopy observations of the transfection efficiency. (**c**) The cell viability of BT474 was detected via MTT. *** *p* < 0.001 vs. pcDNA3.1-EGFP.

To confirm that LP-scFv enhances scFv’s internalization and subsequent anticancer activity, BT474 cells were transiently transfected with pcDNA3.1-scFv-EGFP, and cell viability was assessed. BT474 cells transfected with pcDNA3.1-scFv-EGFP showed significantly reduced viability compared to those transfected with pcDNA3.1-EGFP (Figure 4a–c), suggesting that the scFv inhibited cell proliferation post entry. Previous studies have reported that HER2 is expressed not only on the cell membrane but also in the nucleus and cytoplasm [32,33]. To confirm scFv’s interaction with cytoplasmic HER2 post transfection, thereby regulating epithelial cell proliferation and survival, HER2 protein levels in the membrane and cytoplasmic fractions of BT474 and MCF-7 tumor cell lines were measured, revealing full-length HER2 (185 kDa) in both the cell membrane and cytoplasmic fractions from BT474 cells, whereas only minimal membrane HER2 was detectable in MCF-7 cells (Figure S5). Therefore, LP-scFv may enhance the anti-proliferative effect of the scFv by increasing its intracellular dose through improved internalization.

### 2.4. Mechanism of LP-scFv and scFv Inhibiting the Proliferation of HER2-Positive BT474 Cells

**Figure 5:**
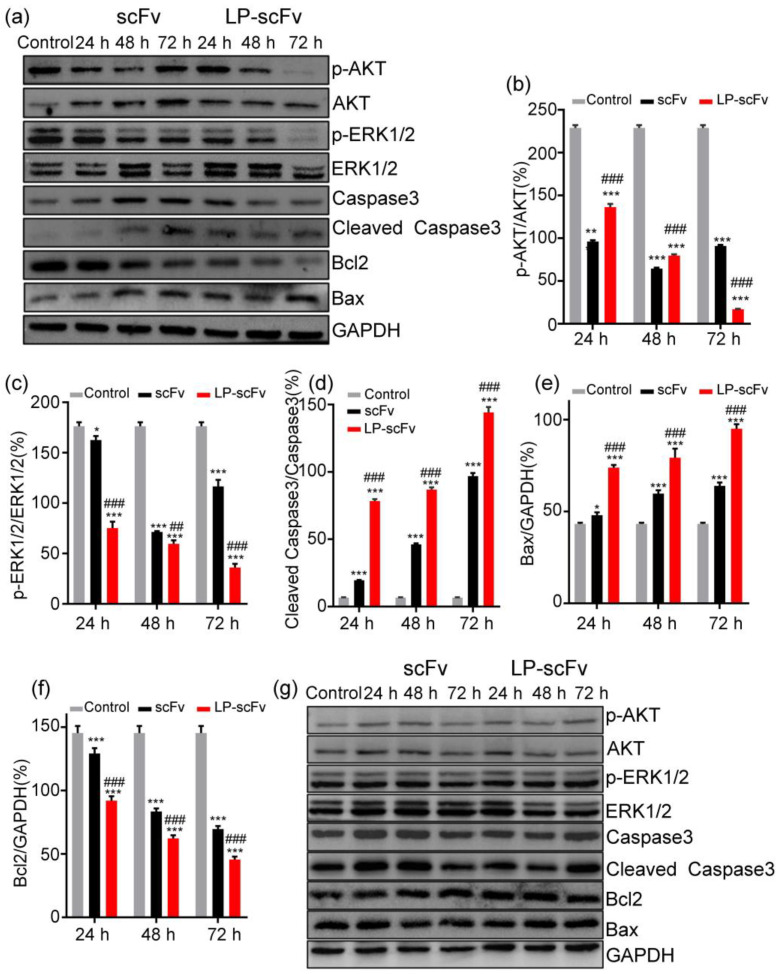
Effects of scFv and LP-scFv on apoptotic signaling pathways. Western blotting was used to measure levels of Caspase-3, Bax, Bcl2, AKT, ERK1/2, p-AKT, ERK1/2, and p-ERK1/2 in BT474 (**a**) and MCF-7 (**g**) cells after treatment with scFv or LP-scFv for 72 h, with GAPDH used as a loading control. Quantitative analysis of the protein expression levels of p-AKT (**b**), ERK1/2, and p-ERK1/2 (**c**) Caspase-3 (**d**), Bax (**e**), and Bcl2 (**f**) protein levels in control, scFv, and LP-scFv group of BT474 cells. * *p* < 0.05, ** *p* < 0.01, *** *p* < 0.001, vs. control; ^##^ *p* > 0.01; ^###^ *p* < 0.001, vs. scFv group.

Monoclonal antibodies targeting the HER2 receptor, including trastuzumab (Herceptin) and pertuzumab (Perjeta), inhibit downstream HER2 tyrosine kinase signaling via the MAPK-ERK1/2 and PI3K/AKT pathways, resulting in cell apoptosis and proliferation arrest [34,35]. Considering that LP induction promoted scFv internalization and antitumor activity in HER2-positive cells in vitro, the mechanisms were further analyzed through the MAPK-ERK1/2 and PI3K/AKT pathways. The expression levels of apoptosis-related proteins, such as Caspase-3, cleaved Caspase-3, Bax, and Bcl2, were also investigated via Western blotting. The results indicated that BT474 cells treated with the scFv and LP-scFv exhibited significantly increased expressions of Bax and cleaved Caspase-3 and a significant decrease in Bcl2 levels compared to the control group. Notably, these effects were more pronounced with LP-scFv than with the scFv alone (Figure 5a–f). BT474 cells treated with the scFv and LP-scFv showed significant reductions in phosphorylated ERK1/2 (p-ERK1/2) and AKT (p-AKT) levels, with LP-scFv having a stronger effect than the scFv. In line with the flow cytometry results, neither the scFv nor LP-scFv induced apoptotic death or significant changes in AKT or ERK phosphorylation in MCF-7 cells (Figure 5g). These findings indicate that LP-scFv enhances the cytotoxic activity and apoptosis of the scFv in BT474 cells through the MAPK-ERK1/2 and PI3K/AKT pathways. Collectively, LP introduction promotes scFv internalization without altering its mechanism of action, improving the scFv’s anticancer activity in HER2-positive BT474 cells by increasing its intracellular dosage.

### 2.5. Detection of scFv and LP-scFv’s Antitumor Activity In Vivo

**Figure 6:**
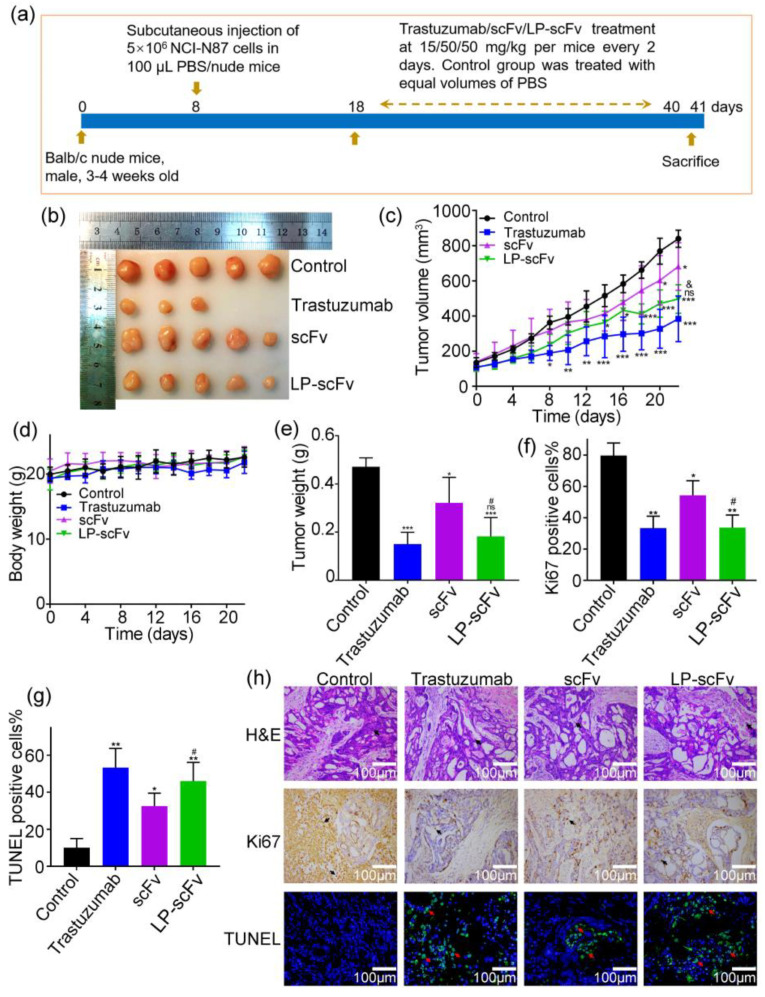
In vivo antitumor efficacy of scFv and LP-scFv in NCI-N87 xenograft model mice. (**a**) Schematic diagram of the experimental protocol. (**b**) Images of collected tumors. The changes in tumor volume (**c**), tumor weight (**d**), and body weight (**e**) were monitored with the treatment time. Ki-67-positive (**f**) and TUNEL-positive (**g**) cells were counted in randomly acquired images. (**h**) H&E, anti-Ki-67 antibody staining, and TUNEL staining (200×) of tumors after treatment with different formulations (Scale bar, 100 μm). * *p* < 0.05, ** *p* < 0.01, *** *p* < 0.001, vs. control; ^#^ *p* < 0.05, ^&^ *p* < 0.05, vs. scFv group; ns, no significance, vs. trastuzumab group.

HER2 is a therapeutic target in both breast cancer and certain gastric tumors [36]. The HER2-positive gastric cancer cell line, NCI-N87, serves as an effective model for HER2-targeting therapy in vivo [37,38]. To assess LP-scFv’s in vivo antitumor activity, mice bearing NCI-N87 tumor xenografts were used as models (Figure 6a). Figure 6b,c show that subcutaneous injections of trastuzumab, the scFv, or LP-scFv every other day significantly reduced the NCI-N87 tumor weight and size compared to the PBS-treated control group. Tumor growth inhibition (TGI%) was 55.7% for trastuzumab, 23.1% for scFv, and 45.2% for LP-scFv. No body weight loss was observed in any murine treatment group (Figure 6d). In the trastuzumab, scFv, and LP-scFv groups, tumor weights decreased by 68.1%, 31.8%, and 61.4%, respectively, compared to the control group (Figure 6e). These results indicate that LP-scFv significantly improved scFv’s TGI rate. While LP-scFv’s tumor suppression was not greater than that of trastuzumab, the difference was not statistically significant.

Ki-67, a nuclear DNA-binding protein associated with the cell cycle, is widely utilized as a marker of cell proliferation [39]. Furthermore, the TUNEL assay is employed to evaluate apoptosis by labeling the exposed termini of DNA and visualizing nuclei with fragmented DNA [40]. Ki-67 expression was reduced in the trastuzumab, scFv, and LP-scFv groups compared to the control group. TUNEL staining assays demonstrated that trastuzumab, scFv, and LP-scFv effectively induced cellular apoptosis (Figure 6f–h). LP-scFv inhibited tumor cell proliferation and enhanced apoptosis more significantly compared to scFv, aligning with our in vitro findings.

### 2.6. Safety Evaluation of scFv and LP-scFv In Vivo

**Figure 7:**
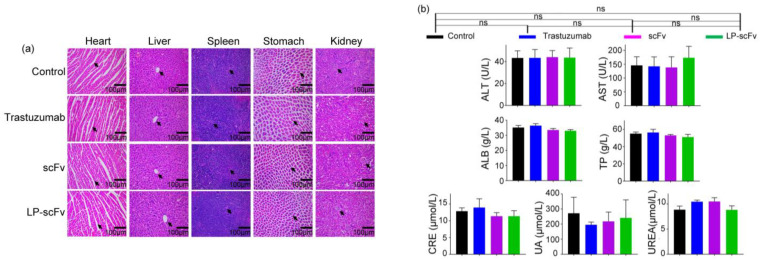
The systemic toxicity of trastuzumab, scFv, and LP-scFv in tumor-bearing mice. (**a**) H&E staining images (200×) for major tissues (heart, liver, spleen, stomach, and kidney) from the mice treated in the control group (PBS) and treatment groups (trastuzumab, scFv, and LP-scFv) (Scale bar, 100 μm) and the identification characteristics were pointed with an arrow. (**b**) Liver function indices (ALT, AST, TP, and ALB levels) and kidney function indices (CRE, UA, and UREA levels). Data are represented as the means ± SD; ns, no significance.

Following the animal experiments, major organs such as the heart, liver, spleen, stomach, and kidneys were collected for histopathological evaluation using hematoxylin and eosin (H&E) staining. Additionally, serum samples from the control and experimental groups were analyzed for liver and kidney function tests. Figure 7a indicates no significant morphological differences in the trastuzumab, scFv, and LP-scFv groups compared to the control group. Similarly, Figure 7b shows no significant changes in liver function indicators (alanine aminotransferase [ALT], aspartate aminotransferase [AST], total proteins [TP], albumin [ALB]) or renal function biomarkers (creatinine [CRE], blood urea [UREA], blood uric acid [UA]) in these treatment groups. These results suggest that the scFv and LP-scFv demonstrated effective therapeutic efficacy without significant toxicity to major organs in mice.

## 3. Materials and Methods

### 3.1. Expression and Purification of LP-EGFP, scFv, and LP-scFv

The LP-EGFP, scFv, and LP-scFv genes were cloned between the *BamH*I and *Nde*I sites in the pET28a (+) vector with appropriate primers (Table S1). Competent *E. coli* BL21 (DE3) (Tiangen, Beijing, China) cells were transformed using these plasmids, after which cells were collected via centrifugation at 6500 rpm for 10 min at 4 °C. The cell pellets were lysed, supernatants were then loaded onto a Ni-NTA agarose column (GE Healthcare Life Sciences, Uppsala, Sweden), and bound protein was eluted with different concentrations of imidazole. SDS-PAGE and Western blotting were used to confirm expression and purity. Proteins were then dialyzed against PBS, concentrated to 0.5 mg/mL, and stored at −80 °C.

### 3.2. Assessment of scFv and LP-scFv HER2 Affinity

The affinity of the scFv and LP-scFv for HER2 was assessed using biolayer interferometry (BLI) with a ForteBio Octet RED96 system (Pall ForteBio, Fremont, CA, USA). For the analysis, both antibodies and the HER2 antigen were diluted in PBST buffer. Anti-human IgG Fc (AHC) biosensors were equilibrated in PBST for 10 min and then washed in PBST for 1 min to establish a baseline. Subsequently, recombinant human HER2 antigen (10 μg/mL, ab168896, Abcam, Cambridge, UK) was applied to the sensors for 100 s. The sensors were rinsed in a kinetics buffer for 1 min before being immersed in solutions with varying concentrations (100, 200, 300 nM) of the scFv or LP-scFv for a 160 s association step. The biosensors were then placed back into the assay buffer for a 160 s dissociation step. The resulting data were analyzed using a 1:1 Langmuir binding model with ForteBio 9.0 data analysis software.

### 3.3. Cell Culture

MCF-7, BT474, NCI-N87, and MCF-10A cells were grown in RPMI-1640 (Gibco, Billings, MT, USA), DMEM (Gibco, USA), RPMI-1640 (Gibco, USA), and an MEGM Bullet Kit (Lonza, Walkersville, MD, USA), respectively. Culture media for the MCF-7, BT474, and NCI-N87 cells were supplemented with 10% FBS (Gibco, USA) and a 1% penicillin and streptomycin solution (Gibco, USA). The culture medium for the MCF-10A cells was supplemented with 100 ng/mL cholera toxin (Sigma, St. Louis, MO, USA). Cells were grown in 5% CO_2_ at 37 °C, with 95% humidity.

### 3.4. Binding and Internalization Analysis

A total of 5 × 10^3^ cells were seeded into 35 mm glass culture dishes (NEST, Beijing, China) for 24 h, after which they were treated with Hoechst 33342 (2 μg/mL; Invitrogen, Waltham, MA, USA) for 30 min at 37 °C. Cells were then incubated with either scFv-EGFP or LP-scFv-EGFP (30 μg) for 30 min at 4 °C, followed by washing with ice-cold PBS to remove unbound substances. After washing, the cells were incubated at 37 °C for 2 h to facilitate internalization. Subsequently, cells were examined using confocal laser scanning microscopy (600× magnification; Leica Microsystems, Wetzlar, Germany). BT474 cells treated with scFv-EGFP and LP-scFv-EGFP at 4 °C for 30 min were subsequently incubated at 37 °C for 2 h. Hoechst 33342 and DiD far-infrared were used to stain the nucleus and cell membrane, respectively. Imaging was performed using a laser confocal microscope equipped with a 63× (NA 1.46, Oil) objective lens and argon lasers (405 nm, 488 nm, and 650 nm). The image acquisition parameters were set as follows: x: 90 μm, y: 90 μm, z-axis step length: 0.54 μm, image size: 1024 × 1024. A total of 40 cell images were captured and stacked to create 3D cell images.

The binding and internalization of these fragments were further analyzed via flow cytometry, with the cells being processed as above. FlowJo vX software was used to determine the geometric mean fluorescence intensity for each sample, and the degree of internalization for these proteins was calculated based on the following formula [30,31]:

(1) $$\mathrm{Internalized}\%=\frac{\mathrm{Total}\;\mathrm{surface}-\mathrm{bound}\;(4\;{}^{\circ}C)\;-\mathrm{Total}\;\mathrm{surface}-\mathrm{bound}\;(37\;{}^{\circ}C)}{\mathrm{Total}\;\mathrm{surface}-\mathrm{bound}\;(4\;{}^{\circ}C)\;}\times 100\%$$

### 3.5. MTT Assay

Following overnight incubation in 96-well plates (5 × 10^3^/well), BT474, NCI-N87, MCF-10A, and MCF-7 cells were treated with various concentrations of the scFv or LP-scFv (0–1000 nM) for 72 h. MTT (20 μL/well, 5 mg/mL) was then added, and the plates were further incubated at 37 °C for another 4 h, after which DMSO (150 μL) was added in each well for 10 min to dissolve the formazan crystals. The absorbance at 490 nm [41] was measured using a SpectraMax190 microplate reader (Molecular Devices, Sunnyvale, CA, USA).

### 3.6. Measurement of Cell Apoptosis

BT474 and MCF-7 cells were seeded in 6-well plates and incubated overnight. Subsequently, the cells were treated with 500 nM of the scFv or LP-scFv for 72 h. Untreated cells served as controls. For the apoptosis analysis, a Cell Apoptosis Kit containing Annexin V AF488 and Propidium Iodide (PI) (Invitrogen, USA) was employed, with flow cytometry used to assess cell apoptotic death.

### 3.7. Western Blotting

Cells were lysed using RIPA buffer supplemented with 1 mM PMSF and protease inhibitors (WB0122, Shanghai Wei AO Biological Technology, Shanghai, China). Protein extracts were quantified using a BCA assay. Subsequently, proteins were separated using SDS-PAGE and transferred onto PVDF membranes (Millipore, Temecula, CA, USA). The membranes were blocked and subsequently incubated overnight with primary antibodies targeting AKT (#9272), ERK1/2 (#4370), Bax (#2772), Bcl2 (#2870S), p-AKT (#4060), p-ERK1/2 (#4695), Caspase3 (A2156, ABclonal Technology, Woburn, MA, USA), and GAPDH (#2118). Unless specified otherwise, all primary antibodies, sourced from Cell Signalling Technology (Danvers, MA, USA), were used at a 1:1000 dilution. Following this, the membranes were incubated with HRP-conjugated goat anti-rabbit secondary antibody (1:5000; Ab6721, Abcam, Waltham, MA, USA) at room temperature for 1 h and then developed using an enhanced chemiluminescence substrate (ECL, Thermo Fisher Scientific, Carlsbad, CA, USA).

### 3.8. Cell Transfection

The pComb3HSS-scFv plasmid was utilized for the amplification of HER2 scFv, employing the primers detailed in Table S2. Subsequently, scFv genes were cloned between the *AfIII* and *AgeI* sites of the pcDNA3.1 (+)-EGFP vector. For transfection, 10,000 BT474 or MCF-7 cells were seeded per well in a 96-well plate and allowed to adhere overnight. A solution of 0.1 μg of plasmid DNA in 10 μL of medium was prepared and briefly vortexed. Separately, 0.4 μL of GenJet reagent (SL100489, SignaGen Laboratories, Frederick, MD, USA) was mixed with an additional 10 μL of media for each well. These solutions were then gently combined through pipetting and left to rest for 15 min. The mixture was then added to the cells in the 96 well plates. After 4–6 h of incubation, the mixture was replaced with fresh growth media. Cells were further incubated at 37 °C for 48 h. Post incubation, green fluorescence was observed using a fluorescence microscope to assess the transfection efficiency.

### 3.9. Assessment of Antitumor Effect in Xenograft Model Mice

The effect of the scFv and LP-scFv on tumor growth was assessed using tumor xenograft model mice. All animal procedures and studies were approved by the Institutional Animal Care and Use Committee of South China University of Technology (Guangzhou, China, Ref: 20180009, Date: 18 December 2019). Female BALB/c nude mice (3–4 weeks old, 16–18 g) were obtained from Hunan Slack Jingda Laboratory Animal Co., Ltd. (Changsha, China) and housed in a pathogen-free environment. The dorsal flank of each mouse was subcutaneously inoculated with 5 × 10^6^ NCI-N87 cells. When tumor volumes reached 100–150 mm^3^, the nude mice were randomly divided into four groups for subcutaneous treatment [42,43,44], approximately 1 cm away from the tumor site. Details of the animal groups and treatment protocols are provided in Table 2.

Tumor size and body weight were measured during this period. The tumor volume was measured with calipers and was calculated as V = 0.5 × length × (width)^2^. All mice were sacrificed on the 21st day after tumor implantation, and tumor tissues were collected, weighed, and imaged. The TGI% was calculated using the following formula.

(2) $$\mathrm{TGI}\%=\left(1-\frac{V_{\mathrm{treated}}\left(d\right)-V_{\mathrm{treated}}\left(0\right)}{V_{\mathrm{control}}\left(d\right)-V_{\mathrm{control}}\left(0\right)}\right)\times 100\%$$

Tumors and major organs, including the heart, liver, spleen, stomach, and kidneys, were collected for histopathological examination through H&E staining. The tumor inhibition rate was calculated according to the average tumor weight (W). TUNEL and Ki67 staining was performed in the selected organs to assess efficiency. Following the TUNEL detection kit’s (G1506, Servicebio, Wuhan, China) instructions, the tissue sections were treated with a detection mixture (TDT enzyme, dUTP, and buffer mixed in a 1:5:50 ratio) to perform the labeling. The sections were incubated for 1 h at 37 °C, and then washed 4 times with PBS (pH = 7.4) for 5 min each time. Then, the sections were added to 100 μL of click reaction solution, incubated for 30 min at room temperature, and washed with PBS twice for 5 min each time. Sections were counterstained with Hoechst 33258 for 8 min at room temperature and finally observed under a fluorescence microscope. For Ki-67 immunochemistry, the sections were placed in 0.01 M citrate buffer (pH 6.0) at 100 °C for 10 min for antigen repair. After blocking in 5% sheep serum, sections were incubated with rabbit monoclonal antibody Ki-67 (GB151499, Servicebio, China) at a dilution of 1:500 overnight at 4 °C. Subsequently, the goat anti-rabbit lgG (H+L) secondary antibody conjugated with HRP (1:200; GB23303, Servicebio, China) was applied at room temperature for 40 min. Sections were stained with DAB, then counterstained with hematoxylin, dehydrated, and fixed.

(3) $$\mathrm{Tumor}\;\mathrm{inhibition}\;\%=\left(1-\frac{W_{\mathrm{treated}}}{W_{\mathrm{control}}}\right)\times 100\%$$

### 3.10. Statistical Analysis

The presented results are representative of three or more independent experiments, with data expressed as means ± standard deviation (SD). All statistical analyses were conducted using GraphPad Prism 7.0. Comparisons between two groups were made using *t*-tests, while comparisons involving more than two groups used two-way ANOVAs. A *p*-value < 0.05 was considered statistically significant.

## 4. Conclusions

In conclusion, this study highlights the effects of enhancing antibody endocytosis. Both the scFv and the LP are small-protein molecules; the LP, an oligopeptide carrier, effectively traverses cell membranes to deliver substances, while the scFv exhibits antitumor activity. We hypothesized that a fusion molecule combining an LP and scFv could yield improved characteristics. To test this, a novel antibody-based drug, LP-scFv, was engineered by fusing an anti-HER2 scFv with an LP. This fusion enhances membrane penetration and protein transport, thereby increasing the effective internalization and subsequent antitumor activity of the scFv.

The principal findings of this study are as follows: Firstly, the LP-scFv conjugate, successfully expressed in the *E. coli* system, significantly enhanced the internalization efficiency of the scFv (approximately twofold) while preserving its cell specificity, affinity for the HER2 antigen, and anticancer activity. LP-scFv augmented the inhibition of phosphorylation in the MAPK-ERK1/2 and PI3K/AKT signaling pathways, demonstrating greater suppression of cell proliferation and promotion of apoptosis in vitro. Secondly, in vivo experiments using a xenotransplantation model confirmed that LP conjugation significantly improved scFv’s antitumor activity, with LP-scFv’s effects comparable to trastuzumab. H&E staining and serum biochemical analyses indicated the low toxicity and good tolerability of both the scFv and LP-scFv. Finally, this study acknowledges certain limitations, such as the need to explore the efficacy of whole antibody conjugated with LP, the effects of LP-scFv in different animal species, and the underlying endocytosis mechanisms of LP-scFv. Overall, our research demonstrates that constructing LP-scFv is an effective strategy to enhance the scFv’s antitumor activity both in vivo and in vitro. As an HER2-positive breast cancer-specific drug or a small-molecule drug carrier model, LP-scFv, with its simple process, holds significant potential for application. These findings offer a novel approach to improving internalization efficiency and serve as a reference for designing new, small antibody drugs.

## 5. Patents

The patent for fusion protein LP-scFv has been granted (CN Patent No. 201710683659.5) (http://epub.cnipa.gov.cn/Index, accessed on 25 March 2022).

## Figures and Tables

**Table 1:** Fitted binding parameters, on-rate (*k*_on_), off-rate (*k*_off_), and *K*_D_ for scFv and LP-scFv.

Antigen	Antibody	*k*_on_ (M^−1^S^−1^)	*k*_off_ (S^−1^)	*K*_D_ (nM)	χ^2^	R^2^
HER2	scFv	(1.05 ± 0.15) × 10^5^	(2.28 ± 0.31) × 10^−3^	21.6 ± 2.1	0.04	0.97
HER2	LP-scFv	(4.49 ± 0.49) × 10^4^	(8.63 ± 2.24) × 10^−4^	19.2 ± 3.4	0.05	0.97

**Table 2:** The animal groups and treatment protocols.

Group Name	n	Injection Interval (Day)	Experiment Time (Weeks)	Treatment
Control	5	2	3	PBS (control, subcutaneous administration)
Trastuzumab	3	2	3	PBS + Trastuzumab (15 mg/kg, subcutaneous administration)
scFv	5	2	3	PBS + scFv (50 mg/kg, subcutaneous administration)
LP-scFv	5	2	3	PBS + LP-scFv (50 mg/kg, subcutaneous administration)

## Data Availability

All relevant raw data in the manuscript are available from the corresponding author upon request.

## Supplementary Materials

The following supporting information can be downloaded at: https://www.mdpi.com/article/10.3390/molecules29061247/s1, Figure S1: Expression, purification, and identification of the LP-EGFP, scFv, LP-scFv, scFv-EGFP, and LP-scFv-EGFP proteins; Figure S2: Representative binding/dissociation curves for interactions between HER2 and scFv or LP-scFv as analyzed via BLI; Figure S3: The ability of LP-EGFP (blue curve) promoting the endocytosis of EGFP (green curve); Figure S4: The effects of the LP alone on the viability of BT474, NCI-N87, MCF-7, and MCF-10A cells; Figure S5: Cytoplasmic HER2 expression in BT474 and MCF-7 cells; Table S1: Primers used for pET28a-LP-EGFP, pET28a-scFv, and pET28a-LP-scFv vector construction; Table S2: Primers used for pcDNA3.1 (+)-scFv-EGFP vector construction.
