# Supplementary material for: A Cell-Penetrating Peptide Improves Anti-HER2 Single-Chain Variable Fragment Internalization and Antitumor Activity against HER2-Positive Breast Cancer In Vitro and In Vivo
Source: Molecules. 2024 Mar 11;29(6):1247. doi: 10.3390/molecules29061247 (PMC10974526; doi:10.3390/molecules29061247)
Supplement: Supplementary file 1 [file molecules-29-01247-s001.zip › molecules-2864313-supplementary.pdf]

*Supplementary information*

*for*

**A Cell−Penetrating Peptide Improves Anti−HER2 Single−Chain Variable Fragment Internalization and Antitumor Activity against HER2−Positive Breast Cancer In Vitro and In Vivo**

Junmin Li 1,2,†, Yanting Zhou 1,†, Zhuowei Su 3, Xue Li 1,4, Lei Zhang 1,5 and Shan Li 1,*

1 MOE International Joint Laboratory for Synthetic Biology and Medicines, School of Biology and Biological En-gineering, South China University of Technology, Guangzhou 510006, China; lijunmin5760@163.com (J.L.); laurachow0828@163.com (Y.Z.); snow0377@163.com (X.L.); lzhang-ce@scut.edu.cn (L.Z.)
2 School of Biology and Food Science, Shangqiu Normal University; Shangqiu 476000, China
3 Guangdong Provincial Hospital of Chinese Medicine, The Second Affiliated Hospital of Guangzhou,
University of Chinese Medicine, Guangzhou 510006, China; szw2003@126.com
4 Nanyang Medical College, Nanyang 473001, China
5 NMPA Key Laboratory for Quality Control of Blood Products, Guangdong Institute for Drug Control, Guang-zhou 510663, China
*Correspondence: lishan@scut.edu.cn; Tel.: +86-3938-0678

## Contents

**Figure.S1**. Expression, purification and identification of the LP-EGFP, scFv, LP-scFv, scFv-EGFP and LP-scFv-EGFP proteins. (a)-(c) LP-EGFP, scFv, LP-scFv, scFv-EGFP and LP-scFv-EGFP proteins were detected via SDS-PAGE. (e)-(g) LP-EGFP, scFv, LP-scFv, scFv-EGFP and LP-scFv-EGFP proteins were confirmed via Western blot.
**Figure S2.** Representative binding/dissociation curves for interactions between HER2 and scFv (a) or LP-scFv (b) as analyzed via BLI.
**Figure S3.** The ability of LP-EGFP (blue curve) promoting the endocytosis of EGFP (green curve).
**Figure S4.** The effects of LP alone on the viability of BT474, NCI-N87, MCF-7 and MCF-10A cells.
**Figure S5.** Cytoplasmic HER2 expression in BT474 and MCF-7 cells.
**Table S1.** Primers used for pET28a-LP-EGFP, pET28a-scFv and pET28a-LP-scFv vector construction.
**Table S2.** Primers used for pcDNA3.1 (+)-scFv-EGFP vector construction.

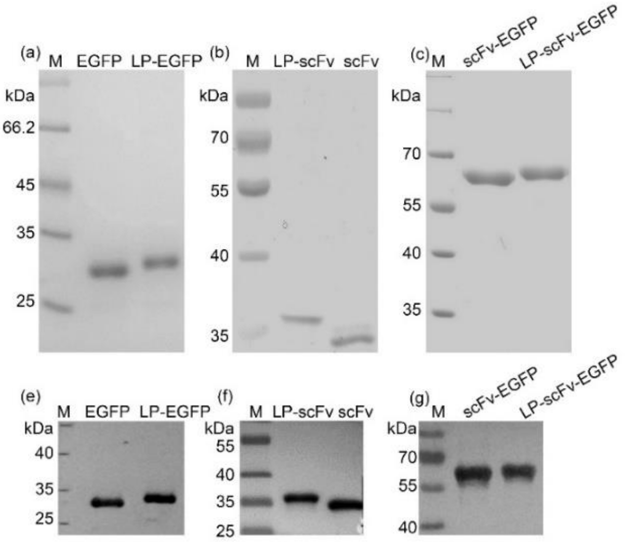


**Figure S1.** Expression, purification and identification of the LP-EGFP, scFv, LP-scFv, scFv-EGFP and LP-scFv-EGFP proteins. (a)-(c) LP-EGFP, scFv, LP-scFv, scFv-EGFP and LP-scFv-EGFP proteins were detected via SDS-PAGE. (e)-(g) LP-EGFP, scFv, LP-scFv, scFv-EGFP and LP-scFv-GFP proteins were confirmed via Western blot.

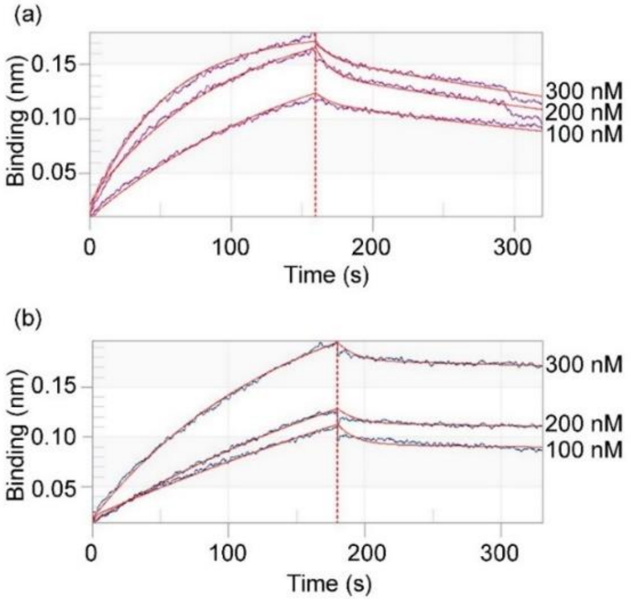


**Figure S2.** Representative binding/dissociation curves for interactions between HER2 and scFv (a) or LP-scFv (b) as analyzed via BLI.

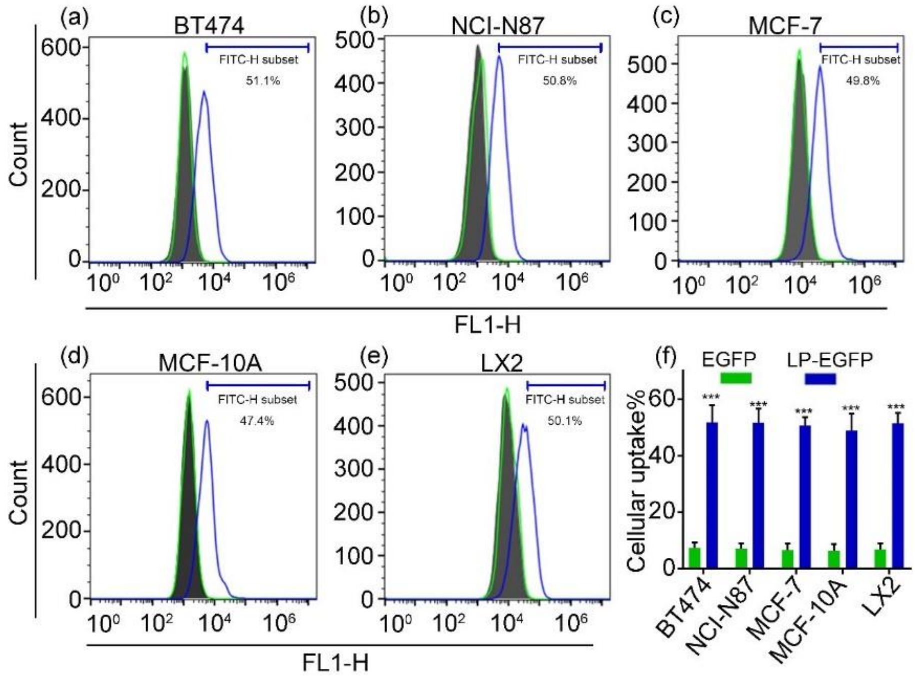


**Figure S3.** The ability of LP-EGFP (blue curve) promoting the endocytosis of EGFP (green curve) into HER2 positive cells BT474 (a), NCI-N87 (b), HER2 negative cells MCF-7 (c), breast normal cells MCF-10A (d) and human hepatic stellate cells LX2 (e) were detected by flow cytometry. (f) The internalization efficacy of LP-EGFP and EGFP in the above cells. ****P* < 0.001, vs. EGFP.

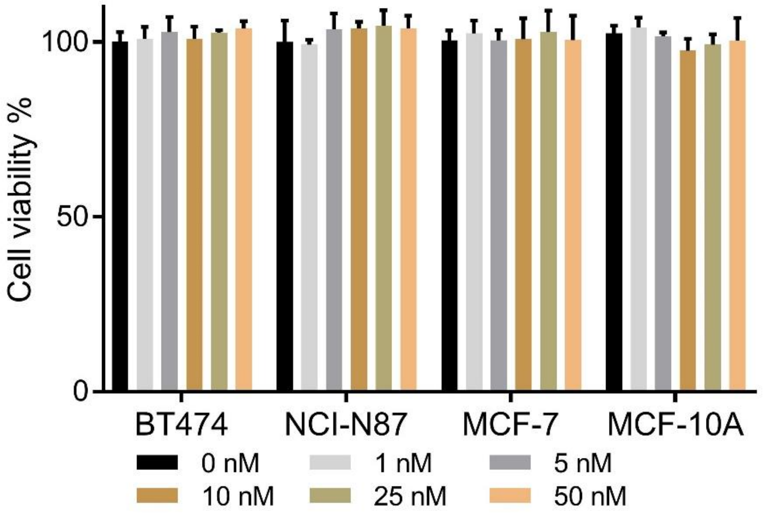


**Figure S4.** The effects of LP alone on the viability of BT474, NCI-N87, MCF-7 and MCF-10A cells.

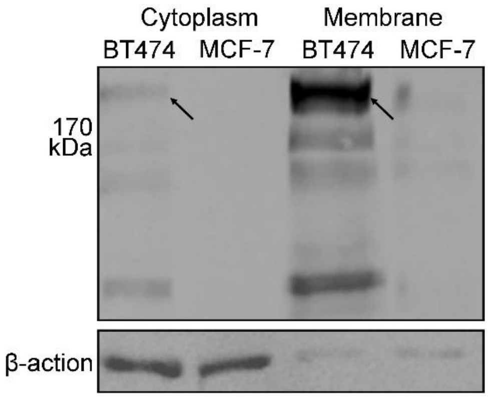


**Figure S5**. Cytoplasmic HER2 expression (black arrow) in BT474 and MCF-7 cells.

**Table S1.** Primers used for pET28a-LP-EGFP, pET28a-scFv and pET28a-LP-scFv vector construction.

| primer | sequence (5′-3′) |
|---|---|
| *LP-EGFP*-F1 | TATTCATATG (*Nde*I) TACCACATGATGCTGTCCGC |
| *LP-EGFP*-R1 | AAGTTCTTCTCCTTTGCTGGATCCGCATGCGCTGCCACCGCCACCATTGT |
| *LP-EGFP*-F2 | ACAATGGTGGCGGTGGCAGCGCATGCGGATCCAGCAAAGGAGAAGAACT |
| *LP-EGFP*-R2 | ATAGAAGCTT (*Hind*III) TTTGTAGAGCTCATCCATGCCA |
| *scFv*-F | GTCTCATATG (*Nde*I) CAAGTACAATTATTACAATCTGGT |
| *scFv*-R | ACGTGGATCC (*BamH* I) TAGATCTCCTCCTAATACTGT |
| *LP-scFv*-F1 | TATTCATATG (*Nde*I) TACCACATGATGCTGTCCGC |
| *LP-scFv*-R1 | AGATTGTAATAATTGTACTTGGCATGCGCTGCCACCGCCACCATTGT |
| *LP-scFv*-F2 | ACAATGGTGGCGGTGGCAGCGCATGCCAAGTACAATTATTACAA |
| *LP-scFv*-R2 | ACGTGGATCC (*BamH* I) TAGATCTCCTCCTAATACTGT |

**Table S2.** Primers used for pcDNA3.1 (+)-scFv-EGFP vector construction.

| primer | sequence (5′-3′) |
|---|---|
| *scFv*-F | GCGTCTTAAG (*Afl*II) GCCACCATGCAGGTCCAACTCCTCCAA |
| *scFv*-R | ATATACCGGT (*Age*I) CGGTGGTGGTGGTGGTGGTGGAATTC |
